# Avian hissing sounds: occurrence, mechanism, ontogeny, function and phylogeny

**DOI:** 10.1098/rsos.251298

**Published:** 2025-11-05

**Authors:** Bert Thys, Marcel Eens

**Affiliations:** ^1^ Deparment of Biology, Behavioural Ecology and Ecophysiology Group, University of Antwerp, Wilrijk, Belgium

**Keywords:** birds, behavioural ecology, evolution, hissing call, function, intraspecific acoustic communication, production mechanism

## Abstract

Sound production is widespread across the animal kingdom and can take many forms and serve various functions. A hissing sound is a non-vocal acoustic signal produced by forced air ventilation and is hypothesized to be a behavioural symplesiomorphy in Amniota. Yet, hissing sounds are typically associated with reptiles and have received comparably little research attention in birds. Here, we identified at least 86 families within 34 avian orders in which members produced hissing sounds. Despite being widespread, almost nothing is currently known about the mechanism(s) of avian hissing sound production. Functions of hissing signals were divided into broad categories based on the social/behavioural context in which they are typically expressed and most evidence pointed towards a main role in threat/defence displays towards predators. Yet, interesting examples exist where avian hissing signals are involved in agonistic interactions, parent-offspring communication, sibling competition, mating displays, pair communication and heterospecific eavesdropping. Our review also emphasizes that research on hissing sounds regarding individual variation, acoustic individuality, geographic variation and fitness consequences is still in its infancy. Overall, hissing sounds are widespread and can be involved in communication in many crucial aspects of avian life, thereby spanning various contemporary disciplines in behavioural and evolutionary ecology.

## 1. Introduction

An acoustic signal is referred to as an acoustic sound emitted by an individual that elicits a response from one or more receivers and that has evolved for this effect [1]. Acoustic signals are widespread across the animal kingdom and, in both vertebrates and invertebrates, they can take various forms and serve a variety of functions [2–5]. In terrestrial vertebrates, vocalizations are typically produced in specialized vocal organs, i.e. the larynx or syrinx, whereby sublaryngeal/subsyringeal pressure from the lungs causes the vibration of mucosal folds to generate a fundamental sound which is then filtered by the upper vocal tract [6]. In birds, vocalizations produced in the syrinx (i.e. songs and calls) are among the most well-studied acoustic signals and represent a model system for understanding the ecology and evolution of communication and animal signals [2].

Birds can also produce non-vocal acoustic signals, i.e. sounds not produced in the syrinx. Examples include the drumming of woodpeckers during territorial behaviour [7,8], the fluttering of feathers by species of snipe (*Gallinago* spp.) during courtship [9], the wing beating of the ruffed grouse (*Bonasa umbellus*) during mating displays [10] and the bill clattering of the white stork (*Ciconia ciconia*) in maintaining pair bonds [11]. Another example of a non-vocal acoustic signal constitutes the hissing sounds emitted by some avian species. Hissing sounds are not produced in the syrinx but instead are produced by forced ventilation, i.e. expelling (or inhaling) air quickly through the respiratory tract [3]. Mechanistic constriction somewhere along the path from lungs to bill then results in turbulent, aerodynamic noises [6]. Yet, hissing sounds are typically associated with reptiles and to some extent with mammals (§2). In birds, with rather few exceptions, hissing sounds have received comparably little research attention and the exact mechanism(s) of their production (i.e. non-vocal and/or vocal) remain largely unknown (§§2 and 4). Also, the potential information conveyed by hissing sounds—especially within intraspecific communication—has been debated and, due to the lack of syntheses on this topic, hissing sounds were actually explicitly omitted from a recent study on the evolutionary origin of intraspecific acoustic communication in choanate vertebrates [12]. As will be outlined below, hissing sounds are widespread across Amniota and believed to potentially serve multiple functions, both in inter- and intraspecific communication, at least in birds (§5) [3,6].

Here, we review the literature (Box 1) on the occurrence of hissing sounds in birds and gather information on the mechanism, ontogeny, function and phylogeny of hissing sound production. In doing so, we aim to: (i) identify avian orders/families and species for which published accounts exist on the production of hissing sounds; (ii) compile the available evidence on the mechanisms and development of hissing sound production; (iii) infer the functions of avian hissing signals based on the social/behavioural context in which they are typically expressed; (iv) highlight the importance of acknowledging repeatability, acoustic individuality and geographic variation of hissing signals within and across free-living avian populations; and (v) summarize the phylogenetic distribution of avian hissing sound production. Whenever applicable, the occurrence of potential sex and age effects on hissing sound production is also discussed. Simultaneously, particular attention is paid to pinpointing current knowledge gaps and providing promising future directions in research on avian hissing sounds. Overall, our review reveals that avian hissing signals are widespread and can be involved in communication in many crucial aspects of avian life, thereby spanning a wide variety of important disciplines in contemporary behavioural and evolutionary ecology.

Box 1. **Literature survey**

A systematic literature search was performed across all years (end date: June 2025) in the ISIWeb of Knowledge database, and via Google Scholar, to assess the state of the art of research on avian hissing sounds. Key papers were identified using a search string that focused on ‘hiss*’, ‘avian’ and ‘bird*’. The ancestry approach (footnote chasing) was applied throughout. Moreover, specific search strings were used to help identify papers within particular avian families, in which ‘hiss*’ was combined with the respective Latin name of avian families, following [13]. A number of studies were identified during opportunistic searches within avian families. Titles, abstracts and full texts were screened for relevance and potential information on the mechanism(s), ontogeny, function and/or phylogeny of hissing sound production. Evidence is referred to as anecdotal when the occurrence of hissing sound production was based on a single descriptive observation. Also, the function described for anecdotal evidence is categorized as 'presumed function’ (see electronic supplementary material, table S1).

## 2. Sound production and evolutionary origin

Methods of animal sound production fall into five main categories: (i) stridulation (the rubbing together of body parts), (ii) vocalization (the vibration of mucosal folds called vocal cords, located in the larynx/syrinx, that extend into the lumen of the respiratory tract), (iii) percussion (the hitting of a body part against a substrate), (iv) forced airflow (production of hissing sounds via forced ventilation), and (v) the tymbal mechanism (via tymbals, i.e. series of external folds on the first abdominal segment that are buckled by internal muscles to produce [substrate-born] vibrations; [3]). For each of these mechanisms, detailed descriptions and their general occurrence across the animal kingdom exist and are not covered in the current review (see [3–6,14–17]). Yet, in the following, a brief overview of the forced airflow mechanism in animals is provided.

Sound production by forced airflow specifically refers to the mechanism whereby a sound is produced by forced ventilation, i.e. expelling (or inhaling) air quickly through the respiratory tract [3,15]. This mechanism can be found in some insects, where air is forced through spiracles. Arguably the best-known example constitutes the Madagascar hissing cockroach (*Gromphadorhina portentosa*), which produces hissing calls via a pair of modified abdominal spiracles. Males of this species can utter different types of hissing sounds during predator disturbance, male-male competition, male courtship and copulation [18,19]. Also, other members of the Blattaria (roaches), as well as some members of the Hemiptera (true bugs), Coleoptera (beetles) and Lepidoptera (moths, butterflies) are known to produce hissing sounds by forcing air through spiracles [3,14,20].

In vertebrates, hissing sound production is not uncommon in Amphibia [4]. For example, the terrestrial salamander *Ensatina eschscholtzii* produces hissing calls accompanied by defensive displays when disturbed [21] and male gladiator frogs (*Hyla faber*) elicit hisses in agonistic interactions with other males [22]. Nonetheless, sound production by forceful airflow is a more common characteristic of the vertebrate taxon Amniota. That is, in many lizards, snakes, turtles, crocodilians, basal birds and basal mammals, both sexes are known to hiss by forced ventilation [15–17,23–25]. Hissing by forced ventilation has therefore been hypothesized to be a basal and primitive sound production mechanism. In fact, many reptiles, if they utter sound at all, only produce hissing sounds when threatened. These sounds are often accompanied by simultaneous visual signals of aggression such as posturing, lunging and mouth-gaping displays [15–17].

**Figure 1.**
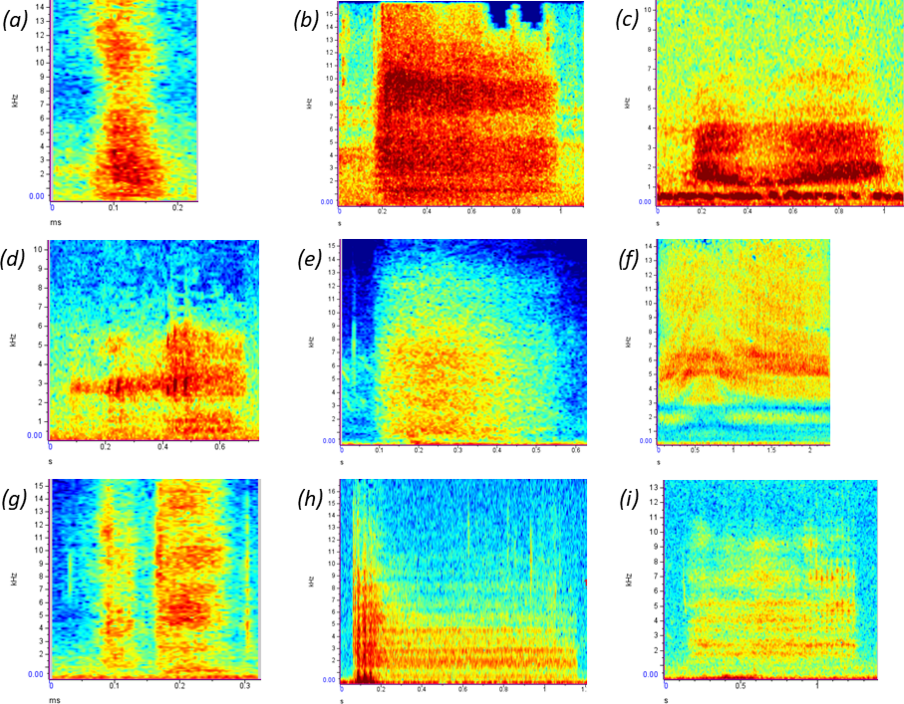
Spectrograms of hissing sounds in birds. (*a*) Common ostrich (*Struthio camelus*) in response to predation threat. (*b*) Canada goose (*Branta canadensis*) in response to predation threat. (*c*) Black grouse (*Lyrurus tetrix*) male during mating displays. (*d*) Eurasian nightjar (*Caprimulgus europaeus*) in response to predation threat. (*e*) Turkey vulture (*Cathartes aura*) during agonistic interaction. (*f*) Burrowing owl (*Athene cunicularia*) in response to predation threat. (*g*) Cockatiel (*Nymphicus hollandicus*) in response to predation threat. (*h*) Great tit (*Parus major*) in response to predation threat. (*i*) Red-billed oxpecker (*Buphagus erythrorynchus*) in response to predation threat. Spectrograms were created using Raven Lite 2.0.5 software (The Cornell Lab, UK). Note that time and frequency axis scales differ between species due to differences in call duration and frequency range, respectively.

In early-branching lineages of birds, such as the common ostrich (*Struthio camelus*; figure 1*a*; electronic supplementary material, video S1), the greater rhea (*Rhea americana*) and geese and swan species (family Anatidae; figure 1*b*; electronic supplementary material, videos S2–S4; see also electronic supplementary material, tables S2 and S3), both sexes also seem to hiss mainly in contexts related to heterospecific and/or conspecific threat (§5). Similarly, in early-branching lineages of mammals, such as marsupials, some species produce hissing sounds during agonistic interactions and in disturbance contexts [24]. Other mammals are also known to produce hissing sounds under threat, including, for example, giraffes (*Giraffa camelopardalis*), musk deer (*Moschus moschiferus*), impalas (*Aepyceros melampus*), llamas (*Lama glama*), sloths (suborder Folivora), skunks (*Mephitis mephitis*), cheetahs (*Acinonyx jubatus*), tigers (*Panthera tigris*) and feral and domestic cats (*Felis catus*; [6,26–30]). Consequently, hissing as a threat/defence display has been proposed to be a behavioural symplesiomorphy for Amniota. If so, the ability to hiss would have been present by the Pennsylvanian period (±320 Ma present) and represents the basal condition for all Amniota [3].

Overall, hissing and other broadband sounds are used in response to predators in many taxa, which is probably because of some general noxious or startling quality inherent in these sounds for many vertebrate species [31–35]. Specifically, based on motivation-structural rules, harsh, abrupt and broadband sounds fit the structure of aggressive signals in vertebrates and contain characteristics that are inherently frightening or arousing [31]. Hence, the general occurrence of broadband sounds in contexts where animals are being threatened and/or are threatening another has been argued to be best explained by evolutionary convergence [31,32].

## 3. A historical appraisal: early reports in birds

That birds can produce hissing sounds appears in the natural history literature at least as early as the 1800s (e.g. [36–38]) and probably even dates back to Aristotle [39]. The early, mostly anecdotal, descriptions of hissing sounds in birds are relatively scarce, yet widely distributed across avian orders and families. Early reports exist for members of, among others, the Struthionidae (ostriches; [40]), Anatidae (geese, swans and ducks; [41]), Phasianidae (chickens, pheasants and allies; [42]), Caprimulgidae (nightjars; [43]), Ardeidae (herons, egrets and bitterns; [44]), Tytonidae (barn-owls; [38,45]), Picidae (woodpeckers; [46]), Phylloscopidae (leaf warblers; [47]) and Paridae (tits; [37,48]). Yet, detailed descriptions remained scarce, at least up until the second half of the twentieth century.

One of the first detailed sequential and illustrated descriptions of a hissing sound and associated display dates back to 1928 for the hole-nesting Carolina chickadee (*Poecile carolinensis*; family Paridae [48]). This was followed by a detailed account by Hinde for the great tit (*Parus major*), a member of the same family [49]. In both species, when confronted with a human observer or other potential predator at the nest cavity, some adult females give an intimidatory display (electronic supplementary material, video S5). Specifically, the sitting female rises slightly on the legs, while the head is raised to *ca* 60° above horizontal. At times, she sways the head from side to side. Next, as if with great effort, the female thrusts the head strongly forward, affecting the whole body, including wings and tail. The tail is fanned out, and the expanding wings shoot out sideways, striking the inside of the cavity. Simultaneously, as the head comes stiffly down, the female emits an explosive ‘snake-like’ hissing sound while violently snapping shut the beak (figure 1*h*; [48,49]). Given that such a hissing display is omnipresent within the Paridae (electronic supplementary material, table S1), it is probably not surprising that subsequent literature on hissing in birds was, and to a large extent still is, dominated by studies on members of this family (e.g. [50–55]).

Early detailed reports on hissing sound production also exist for other avian species. Eurasian wrynecks (*Jynx torquilla*; family Picidae) produce ‘snake-like’ displays when disturbed at the nest; swaying the head from side to side, erecting the head feathers, darting out the tongue and sometimes producing a hissing sound (electronic supplementary material, video S6 [46]). It is also long known that northern flicker nestlings (*Colaptes auratus*; family Picidae) utter hissing sounds almost constantly during day and night, which has been described as resembling the sound of a hive of bees [50]. Furthermore, geese and swans are well-known hissers in defending their territory, offspring and nest [41]. Another long-known example comes from barn owl nestlings (*Tyto alba*) which produce hissing sounds when disturbed and cornered inside the nest cavity (electronic supplementary material, video S7 [38,45]).

For some families and species research progressed, and hissing sound production, with potential associated visual display, is now well-recognized as part of their behavioural repertoire. This includes the aforementioned family of the Paridae, in which published accounts on hissing sound production exist for at least 17 species (electronic supplementary material, table S1 [50–59]). Further examples of families, discussed more fully below (§5 and §7), in which hissing sounds have received more recent research attention include the Anatidae [23], Phasianidae [60–62], Caprimulgidae [63,64], Strigidae [33,65] and Tytonidae [66]. In contrast, for many families and species, only a single or few primary accounts exist to date, which therefore appear to reside in the realm of anecdotal evidence (electronic supplementary material, table S1 [67]). As a consequence, in a lot of cases, solid experimental investigations of the production mechanism(s) and biological roles of avian hissing sounds are currently lacking.

## 4. Mechanical mechanism and ontogeny

Insights into the production mechanism(s) of hissing sounds can provide valuable clues to their development and evolution. Yet, the exact mechanical mechanism by which avian hissing sounds are produced has to our knowledge only been investigated in one species, the domestic goose (*Anser anser domesticus* [68]). In this species, the sound is produced during a long expiration, lasting multiple seconds and preceded by a deep inspiration. Maximum air sac pressure and air flow rate during hissing are intermediate between those of normal breathing and vocalization. Changes in tracheal pressure are almost identical to changes in air sac pressure, which indicates that syringeal resistance is small. Hence, by comparing maximum pressure and flow excursions during an inspiration and expiration, it was demonstrated that hissing is caused by a stream of expiratory air escaping from the constricted glottis [68]. This mechanism largely corroborates with evidence for hissing sound production in various non-avian reptiles [15,17].

In snakes, it is, however, well-established that species can hiss via a variety of mechanisms. That is, a hiss can be produced during either expiration or inspiration, and the terminal course of the airstream can vary; some hiss while holding the mouth open, others hiss through a closed mouth, and yet others hiss through the nostrils [15]. Interestingly, in birds, ruffed grouse males have been described to produce a double hiss, the first half during an exhale and the second during an inhale [69]. Moreover, nasal hissing also appears to occur in birds, such as, for example, in the hoatzin (*Opisthocomus hoazin* [70]). Altogether however, almost nothing is currently known about the exact (mechanical) mechanisms of avian hissing sound production. Given this lack of information for most avian species, we opted for a broad-sense approach and considered a species to produce hissing sounds when described to do so in the literature. Yet, we acknowledge that whether this is via forced ventilation (or rather vocally, i.e. syringeal involvement) in all cases remains to be established (see below).

Despite poor knowledge about avian hissing sound production mechanisms, spectrographic analyses of hissing sounds are increasingly available for a variety of species (figure 1; electronic supplementary material, table S2; see e.g. [23,25,54,61,71–76]). Avian hissing sounds are typically broadband (i.e. wide frequency range) noises, very similar to white noise, as is the case in snakes, lizards and crocodilians [15,17]. For example, in snakes, hisses generally have a broad frequency span of approximately 3–13 kHz, with relatively limited evidence for temporal patterning, amplitude or frequency modulation or even harmonics [15]. There is, however, surprisingly little direct evidence that avian hissing sounds are spectrographically similar to snake hisses (but see [35,75,77]). This highlights the need for researchers to address this possibility in a variety of avian study systems, especially when aiming to unravel the occurrence of acoustic (snake) mimicry within and across avian species (§5.1). At the same time, investigating acoustic (spectral, temporal, amplitude) features and their variation may help reveal underlying mechanisms contributing to hissing sounds being distinct within and among populations and species (§6), as well as whether hissing sounds of respective avian species are non-vocally and/or vocally produced. For example, recent spectrographic analyses of sound production in the common ostrich revealed that adult males not only produce non-vocal hisses (figure 1*a*), but also so-called ‘tonal hisses’ (i.e. hiss-like vocalizations; see [25, fig. 6]). Although not yet experimentally investigated, the latter have been suggested to be created by two separate sources: one by the vibration of the labia in the syrinx and one by the expulsion of air through the larynx and glottis [25]. Whether this and/or other mechanisms are at play in other avian orders warrants further research, especially given the basal and influential position of the common ostrich in avian phylogeny (§7 [78]).

Poor knowledge about the production mechanisms of avian hissing sounds is translated into poor knowledge about their development. In general, nestling calls are thought to act as important precursors of adult vocalizations (see [72]). In the specific case of the burrowing owl (*Athene cunicularia*; see also §5.1), it has been argued that nestling and adult vocal defensive hissing sounds (i.e. hiss-like vocalizations) represent modified begging calls, where the mimetic hiss of the owl has been derived from the spectrographically similar food-begging call (figure 1*f*; electronic supplementary material, video S8; see [65]). Hiss-like begging calls have been described for a variety of avian species (§5.3) and for some passerines, it is known that nestlings only start producing them at a certain stage during their ontogeny. For example, in blue tits (*Cyanistes caeruleus*), nestling begging calls change from pure tones (i.e. narrowband sounds) at an early age to loud broadband hissing sounds when nestlings are around 12 days old (see [76, fig. 1]). The development of hissing sounds as a threat/defence display has also been described for two members of the Bucerotiformes (hoopoes, wood hoopoes and hornbills). That is, in the Eurasian hoopoe (*Upupa epops*) and common scimitarbill (*Rhinopomastus cyanomelas*), there is evidence that nestlings start responding to threat with hissing sounds only when 10 and 12 days old, respectively, in a similar fashion as to how adults of these species react to threat inside the nest cavity [79,80]. On the other hand, in basal birds like the common ostrich and the greater rhea, juvenile individuals, despite being highly vocal, do not produce hissing sounds, which is in contrast to adults of both sexes in response to threat [25,81]. This suggests either that juveniles of these species can but do not produce hissing sounds in early life, or that they acquire the ability to hiss later in life.

Overall, from a mechanical/mechanistic perspective, almost nothing is known about how birds produce hissing sounds, and conclusive evidence about whether a species produces non-vocal (i.e. forced ventilation) and/or vocal (i.e. syringeal involvement) hissing sounds is lacking in most cases, emphasizing the many opportunities for future research. From an ontogenetic perspective, studies investigating nestling acoustic development indicate that sampling the repertoire of nestlings at a specific age is not likely to give a representative overview of the total repertoire across development. This has important consequences for our understanding of the development of hissing signals in particular and acoustic signal ontogeny more generally. Using a within-subject design, comparative analyses of acoustic ontogeny in species in which both nestlings and adults produce hissing sounds—and species in which either nestlings or adults produce hisses— will prove particularly insightful in improving the understanding of hissing sound production developmental trajectories and age effects.

## 5. Function of avian hissing sounds

**Figure 2.**
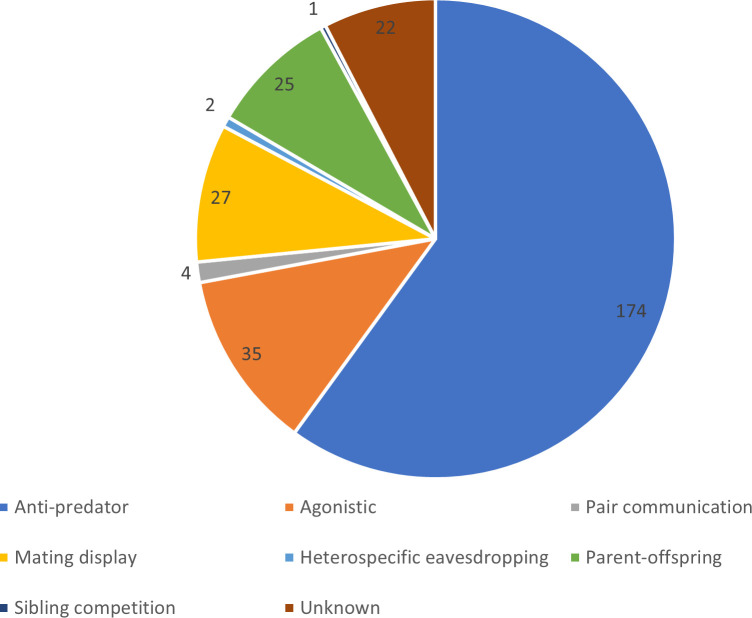
The number of avian species known to produce hissing sounds in different social/behavioural contexts: anti-predator, agonistic interactions, parent-offspring communication (i.e. hiss-like begging calls), sibling competition, mating displays (by males), pair communication (during the breeding season), heterospecific eavesdropping or unknown context. Some studies mention hissing sound production in several contexts for the same species (see electronic supplementary material, table S1).

The function of avian hissing sounds may fall into one of the following categories, related to the social/behavioural context in which they are typically expressed: predator-prey context, agonistic interactions, parent-offspring communication, sibling competition, mating context, pair communication during the breeding season or con- and/or heterospecific eavesdropping (figure 2; electronic supplementary material, table S1). In some cases, social/behavioural contexts can overlap, and hissing sounds may serve multiple functions. It should be noted, however, that in many cases, solid experimental investigations of the biological roles of avian hissing sounds are lacking.

### 5.1. Predator–prey context

Predation is the most common cause of reproductive failure across many avian taxa and a major selective pressure affecting fitness [82,83]. To reduce the consequences of predation, birds have evolved a wide variety of antipredator strategies [84–86]. Interestingly, avian hissing sounds are often part of threat/defence displays in response to potential predators (including humans), as is also the case in reptiles and to a large extent in mammals (§2). Many researchers have hence suggested a role for avian hissing sounds in an anti-predatory context, yet very few have investigated this experimentally. Consequently, the vast majority of quantitative and qualitative evidence comes from a limited number of studies on few, most often hole-nesting species. At the same time, surprisingly few data are available on how potential intruders or natural predators respond to avian hissing sounds.

Arguably the best worked-out example on the function of hissing sounds comes from a series of studies on hole-nesting tit species. Accumulating evidence indicates that tit hissing calls are acoustically similar to snake hisses and prompt similar anxiety and/or vigilance-related responses in a variety of potential (mammalian) nest competitors and predators [34,35,54,56,77]. Moreover, individual variation in hissing behaviour has been shown to come with fitness consequences in terms of reproduction and survival in the wild. For example, in great tits, more fiercely hissing females, compared to less fiercely hissing or silent females, have been shown to initiate clutches later, lay smaller clutches and have lower breeding success [87–90], but are more likely to survive predator attacks [54]. Altogether, this indicates the functional significance of hissing displays in an anti-predatory context in hole-nesting tit species. Other published accounts in hole-nesting species also strongly suggest a role of hissing sounds in an anti-predatory context. For example, a study in barn owls (*Tyto alba*) demonstrated that nestlings can produce extremely noisy hissing calls upon human disturbance at the nest [66]. Similarly, some nestling Eurasian kestrels (*Falco tinnunculus*) produce hissing sounds when captured from the nest and handled by a human observer [91]. Moreover, nestlings and adult females of the Eurasian hoopoe and the common scimitarbill emit loud hissing sounds when confronted with a natural predator inside the nest cavity [79,80].

Other direct experimental evidence for the adverse effects of bird hissing sounds on nest competitors (and potentially predators) comes from studies on burrowing owls. Burrowing owls nest and roost in ground squirrel burrows, the latter also commonly used by rattlesnakes as refugia. When cornered inside their burrow, nestling and adult owls produce a vocal (mimetic) hissing sound which is spectrographically similar to the rattle of rattlesnakes and which elicits similar cautionary responses in mammalian nest competitors ([33]; see also [65]). Consequently, the burrowing owl defensive hiss has been argued to be an example of acoustic Batesian rattlesnake mimicry. Notably, burrowing owls, as well as many other members of the Strigiformes, also produce short non-vocal defensive hisses when threatened (electronic supplementary material, table S1; review in [33]).

In several other avian species, hissing sounds are accompanied by behavioural and/or visual displays that are strongly suggestive of Batesian snake mimicry. The aforementioned anti-predatory hissing display in tit species has long been argued to represent behavioural snake mimicry (electronic supplementary material, video S5; [50]; but see [35,77,90]). Also, the Eurasian wryneck’s defensive display at the nest is described as ‘snake-like’ and, combined with the plumage pattern on the wryneck’s back, has been argued to represent viperid snake mimicry [46,92]. Moreover, in the African cut-throat (*Amadina fasciata*), which lay their eggs in enclosed, ball-shaped nests, individuals perform a ‘snake dance’, which includes writhing the body sinuously, undulating and hissing (electronic supplementary material, video S9 [93]). Surprisingly, we are unaware of any study directly assessing how natural predators respond to these wryneck and cut-throat displays. Further potential examples of mimicry come from the threat/defence displays performed by members of the Caprimulgiformes (nightjars and allies; [64]). Many species of nightjars (Caprimulgidae) and frogmouths (Podargidae) perform behavioural displays towards intruders which consist of wing and tail spreading, mouth gaping, bill snapping, lunging and hissing (figure 1*d* [63,64]). Also, the Australian owlet-nightjar (*Aegotheles cristatus*; Aegothelidae) can produce hissing calls as part of threat displays [36,67]. Whether these latter cases represent examples of behavioural snake mimicry requires further investigation, including as initial steps determining if the produced hissing sounds are spectrographically similar to snake hisses and if they elicit similar adverse responses in natural predators and/or heterospecific nest competitors of the respective species.

Direct experimental evidence for the functional significance of hissing sounds is more scarce in open-nesting and/or ground-dwelling species compared to hole-nesting species. An exception comes from a recent study in the open-nesting zitting cisticola (*Cisticola juncidis* [75]). Nestlings of this species produce hissing sounds when disturbed at the nest by a human observer, which are spectrographically similar to snake hisses (see [75, fig. 2]). Another example comes from Canada geese (*Branta canadensis*), where adults of both sexes are well-known to produce hisses when approached by a human observer at the nest [94]. Also, Indian peahens (*Pavo cristatus*) have been found to produce hissing sounds when confronted with a natural predator at night [95]. Anecdotal evidence for an anti-predatory role of hissing sounds in open-nesting and/or ground-dwelling species also exists for, amongst others, the American bittern (*Botaurus lintiginosus*), the whistling swan (*Cygnus columbianus*), the red-necked grebe (*Podiceps grisegena*) and the kagu (*Rhynochetos jubatus*; see electronic supplementary material, table S1).

Overall, the vast majority of evidence, including anecdotal, indicates that hissing sounds are produced in response to predators and/or heterospecific intruders or competitors (figure 2; see also electronic supplementary material, table S1; §7). This is not surprising given that hissing as a threat/defence display has been argued to be a behavioural symplesiomorphy for Amniota (§2 [3]). Yet, as apparent from the above, the current literature is strongly biased towards few, most often hole-nesting species with very limited solid evidence regarding whether and/or how: (i) avian hissing sounds spectrographically resemble snake hisses; (ii) naturally occurring predators of respective avian species respond to hissing sounds and potentially associated visual displays; and (iii) variation in anti-predatory hissing sound production is linked to life history traits and fitness on the within- and across-population level (see §6). Additionally, as will be outlined in §5.7, very little is known about whether con- and/or heterospecifics eavesdrop on avian anti-predatory hissing signals. Comparative spectrographic analyses, carefully designed playback experiments and long-term population monitoring will be pivotal in elucidating these largely open questions for the variety of avian species that produce hissing sounds in an anti-predatory context.

### 5.2. Agonistic interactions

Competition among individuals for access to limited ecological resources, such as food, nesting space and territories, is widespread across the animal kingdom (competition for mating partners is discussed in §5.5; [93,96]). Such agonistic interactions over limited resources often consist of signalling aggressive intent, which is reflected by ritualized contests and elaborate displays rather than overt and costly fighting [97,98].

Hissing signals can be involved in aggressive interactions among conspecifics (and/or heterospecifics) over limited ecological resources, although direct evidence in birds is rather scarce (figure 2). For example, Eurasian griffon vultures (*Gyps fulvus*) produce hissing sounds during intraspecific competition for access to food [99] and burrow-dwelling little penguins (*Eudyptula minor*) have been reported to hiss when confronted with a conspecific intruder inside their burrow [100]. As a further example, Egyptian geese (*Alopochen aegyptiaca*) occasionally hiss towards greylag geese (*Anser anser*) and *vice versa*, and both species are known to hiss during conspecific aggressive interactions [101–103], most likely as a means of getting access to food and/or territories. In tit species, it has also been argued that hissing displays may be used in defending nesting cavities against conspecific and avian heterospecific nest competitors [35], although data are currently lacking.

Since hissing sounds can be involved in agonistic interactions over limited ecological resources, they are predicted to give a reliable signal of aggressive intent, whereby signalled escalation should indicate a real willingness to fight [97]. Notably, in little penguins, individuals that hiss towards conspecific intruders at their burrow are more likely to attack compared to individuals that growl or bray [100], suggesting that hissing might be a reliable signal of escalated aggression in this species and context. Future research on agonistic hissing sounds should test key game theory predictions regarding the signalling of aggression and competitive ability [98,104]. That is, studies should investigate whether individual variation in agonistic hissing signals reflects competitive ability, i.e. the ability to win or retain access to resources. Also, nothing is currently known about the fitness costs and benefits of individual variation in agonistic hissing displays, as well as whether individual variation is linked to (subjective) resource value and resource holding potential.

### 5.3. Parent-offspring communication

In many species, parents and their dependent offspring are in conflict over the amount of parental care provided, leading to complex family dynamics in terms of, among others, parental food allocation and offspring begging [105,106]. Offspring begging can serve as an honest signal of offspring need, quality or hunger directed to parents [107]. At the same time, begging can serve as a competitive signal among rival siblings for access to food delivered by the parents (i.e. sibling scramble competition; for discussion see §5.4).

In various avian species, nestlings produce hissing sounds while begging for food from their parents, and this hiss-like begging is hence involved in parent–offspring communication (and presumably sibling competition; §5.4). This is, for example, the case in nestlings of the burrowing owl, the little owl (*Athene noctua* [33]), the barn owl [74,108], various lorikeet species [109] and the blue tit [76]. Also, nestlings of the white stork produce quiet hissing sounds during begging displays [11] and, as further examples, hiss-like begging calls are produced by the yellow-billed cuckoo (*Coccyzus americanus*) and the Madagascar cuckoo (*Cuculus rochii* [110,111]), as well as by the citreoline trogon (*Trogon citreolus*), mountain trogon (*T. montanus*) and eared quetzal (*Euptilotis neoxenus*; [112]). Altogether, this illustrates that hiss-like begging calls are rather widespread across birds (figure 2; see also electronic supplementary material, table S1).

Noteworthy is the general assumption of a predation cost of begging. Many studies indicate that conspicuous and exuberant begging can increase predation rates on avian nests (review in [113]; see also [114–116]). Interestingly, there is evidence that the acoustic nature of begging calls, such as frequency range and amplitude, can carry a cost via increased predation [114,117–120]. It could therefore be argued that broadband hiss-like begging calls represent a special case in this context. On the one hand, broadband sounds are theoretically predicted to be easier to locate by potential predators [121,122], although this is not generally supported by empirical evidence of predation rates on avian nests (e.g. [114,118]). On the other hand, broadband sounds have been shown to be rather psychophysically noxious for several vertebrate species, including birds and mammals [31,32,123,124]. As a corollary, it could be hypothesized that hiss-like begging calls may have evolved as a means of reducing predation risk, presumably by being more noxious for avian and mammalian predators. This potentially relaxes predator-induced selection on other aspects of begging, such as begging rate, duration and amplitude, aspects which have been shown to influence predation rates [115,116,119,125]. Yet, whether and how hiss-like begging calls, including their frequency range, amplitude and other features (e.g. call rate, duration, complexity) influence the behaviour of natural predators remains to be established by carefully designed playback experiments.

Overall, avian hiss-like begging calls are rather widespread and expected to adhere to general predictions concerning the evolution of offspring begging [105–107]. Future research should therefore aim to assess: (i) the costs of hiss-like begging (compared to pure tone begging) in terms of energy and/or predation rate; (ii) whether and how hiss-like begging calls honestly signal an offspring’s need, quality or hunger level towards parents; and (iii) the relative role of hiss-like begging in parental food allocation versus sibling scramble competition (§5.4).

### 5.4. Sibling competition

Sibling competition refers to the rivalry among siblings over access to limited parental resources and entails interactions between siblings that increase the fitness of an individual offspring at the expense of the fitness of its siblings [126]. Many studies indicate that offspring can adjust their begging behaviour not only to their own level of need, quality or hunger, but also in response to the number of siblings, the siblings’ behaviour and their size (e.g. [127,128]). Under certain conditions, sibling competition and its associated costs can be reduced by sibling negotiation, whereby nestlings communicate their willingness to compete for impending food to siblings and hence can optimize investment into sibling competition [129,130].

In at least one specific case, experimental evidence exists that hissing sounds are directly involved in sibling competition and negotiation (figure 2). That is, nestlings of the barn owl not only produce hiss-like begging calls for food from their parents but also use these calls (referred to as negotiation calls) among each other in the prolonged nocturnal absence of parents [108]. During parent absence, each nestling acoustically informs its siblings about the willingness to compete over the impending non-divisible food item that parents will deliver, with the hungriest individual producing more and longer calls, thereby deterring siblings momentarily from begging conspicuously at the arrival of the parents [108,128,131]. Nestlings hence use hissing signals to negotiate priority of access to food before parents actually return with a prey item, a process that is believed to reduce the level of sibling competition [129]. It has also been shown that acoustic features of hissing negotiation calls not only reflect hunger level, but also sex and age, as well as individually distinctive characteristics (see §6.1 [74]).

Since hissing sounds are used as food-begging calls in various avian species (§5.3), it is very likely that hissing signals are, at least to some extent, also involved in sibling competition and/or negotiation. Future work should be designed to explicitly address this possibility in species that are known to produce hiss-like begging calls. Nonetheless, overall, it is apparent that hissing sounds can be involved in communication in complex family dynamics, i.e. parent-offspring communication (§5.3) and sibling competition (§5.4), both of which are crucial and integral parts of avian family life.

### 5.5. Mating context

Same-sex individuals often intensely compete for access to mating partners and other limited resources (for the latter see §5.2 [96]). In the case of competition for mates, sexual selection theory predicts that conspicuous traits and elaborate displays evolve by means of intrasexual (same-sex competition) and/or intersexual (mate choice) selection. In other words, variation in the expression of phenotypic traits involved in mating is expected to result in differential mating and/or reproductive success, ultimately via differential success in attracting mates and/or in the ability to outcompete same-sex rivals for access to mates [96].

The production of hissing sounds within a mating context has been described in a few avian species (figure 2). For example, the black grouse (*Lyrurus tetrix*) is a lekking species from the family Phasianidae with intense male–male competition and female choice, resulting in highly skewed annual mating success with few dominant older males monopolizing the vast amount of copulations (see [60]). During displays at leks, males produce two main long-distance sounds, rookooing and hissing (figure 1*c*; electronic supplementary material, video S10). Hissing calls appear to be only produced when there are males, but not females, on the lekking arena [132], suggesting that they are mainly directed at other males. A recent study also revealed individuality in acoustic features of black grouse hissing calls [61], suggesting that competing males and mate-seeking females may be able to recognize and potentially assess males based on individuality of hissing calls (see §6.1). Notably, also in other members of the Phasianidae, such as the ruffed grouse [42,69] and tragopan species (*Tragopan* spp. [133]), males are known to produce hissing calls during mating displays at leks.

In the black-crowned night heron (*Nycticorax nycticorax*), males perform a ‘snap-hiss’ ceremony during pair formation [134]. This ceremony consists of a queer sort of courting dance on the spot of the future nest. With the head and wings lowered, the male treads from one foot to the other with peculiar weaving action. From time to time, he suddenly lowers the head and neck vertically, while his shoulders lift as if in a hiccough and utters a hissing ‘courting cry’. This cry is very deep and quite low and sounds like ‘steam escaping through the safety valve of a boiler’ [134].

In the greater lophorina (*Lophorina superba*), males have been described to occasionally produce a hissing sound during their ‘high intensity display’ [135]. That is, males thrust forward and fully expand the breast shield, flick the cape forward and spread it laterally to form a complete semi-circle over the head and down either side. Below the outer lower edge of the breast shield, black feathers extend inwards and round, forming a complete circle. Just below the central part of the cape, above eyes and bill, two very conspicuous blue-green iridescent ‘eye spots’ appear. Occasionally, with a widely gaping beak, a hissing sound is produced as the male sways from side to side. In this remarkable posture, the male proceeds in short steps along a perch or across a large log, sometimes also dancing around the female [135].

Hence, in a few avian species, male hissing signals are specifically expressed within a mating context (figure 2; see also electronic supplementary material, table S1), strongly suggesting they may be under sexual selection. However, it largely remains to be investigated whether hissing signals play a direct role in same-sex competition and/or mate attraction, as predicted by sexual selection theory. Along this line, it still remains unclear whether individual variation in the expression of such hissing signals results in differential mating and/or reproductive success. To date, we are also unaware of any study reporting on hissing sounds produced by females within a mating context. Investigation of sex effects, seasonal variation, (neuro)physiological influences and fitness consequences of hissing sound production within a mating context will be useful in revealing their occurrence, functional significance and underlying proximate mechanisms (§6.2).

### 5.6. Pair communication during the breeding season

In a few specific cases, hissing sounds have been described to be involved in communication between male and female partners during the breeding season (figure 2; excluding the case of mating displays; §5.5). For example, gentoo penguins (*Pygoscelis papua*) perform a ‘bow-gape-hiss’ display during nest relief ceremonies [136]. Specifically, the individual bends down to the nest, opens its bill showing the bright red lining, and produces a hissing sound. This movement is derived from nest building behaviour, as gentoo penguins may deposit a pebble on the nest and then open their bills and hiss. This ‘bow-gape-hiss’ display is believed to have an appeasement function and a higher display rate by the arriving individual is predictive of a quicker changeover at the nest, i.e. higher speed of nest relief [136].

Hissing sound production is also found during nesting in the parasitic jaeger (*Stercorarius parasiticus*; [137]). Here, the male walks around at parts of the territory with high vegetation cover and occasionally performs nest building movements. When the female is present, she follows the male and, when standing together, both produce so-called ‘squeaking calls’. These calls are mostly bisyllabic, with the first part consisting of a harsh hissing squeak and the second part lacking the squeak, but the hissing remains. Repetition of this ‘squeaking scene’ at a particular location has been observed to determine the ultimate nest site [137].

A final anecdotal example of hissing sound production in pair communication comes from the writhe-billed hornbill (*Rhabdotorrhinus waldeni*; [138]). In this species, females seal themselves inside the nest cavity during nesting, leaving only a narrow slit through which the male can deliver food to the female and offspring. During this sealed-in phase of nesting, females have been observed to produce a subdued hissing sound in response to male food delivery, yet the exact function of this hissing signal is currently unknown [138].

Overall, in a few avian cases, hissing signals can be part of pair communication during the breeding season (figure 2). Whether such hissing signals are, as a general rule, involved in rather formalized interactions and part of more elaborate displays, as is the case in gentoo penguins and parasitic jaegers, requires further research attention. Also, we are unaware of any studies reporting on hissing sound production in pair communication outside a breeding context. This may be due to the fact that in many avian species, sexes mainly interact during the breeding season and/or because it is (logistically) harder to investigate such interactions outside the breeding season. Further research may reveal more cases in which hissing signals are used during avian pair communication in, and potentially outside, a breeding context.

### 5.7. Conspecific and/or heterospecific eavesdropping

One important aspect of animal communication is the transmission of risk-related information among conspecifics and, in some cases, also heterospecifics, as they can eavesdrop on this crucial information [85,139]. Acoustic alarm signals produced by an individual are publicly available and can be used by con- and/or heterospecifics to detect and appropriately respond to immediate predation threat (e.g. by crypsis, becoming vigilant, mobbing the predator, fleeing for cover). Besides the positive effects for immediate danger assessment, eavesdropping on con- or heterospecific alarm calls can also result in indirect and longer-term benefits. These include increased foraging success due to the decreased need for vigilance, adjustment of habitat selection and breeding behaviour based on dynamic spatial information on predation risk, and the facilitation of social learning about previously unknown predator signals. As such, anti-predatory alarm calls can convey critical, fitness-related information for both conspecific and heterospecific receivers [85,139]. Eavesdropping on con- and/or heterospecific acoustic signals can also occur in other behavioural contexts and mediate a variety of social behaviours, including resource defence, aggressive contests and mate attraction [140].

Anti-predatory hissing calls (§5.1), like other alarm calls, are very likely to give risk-related information to con- and/or heterospecifics. To date, we are, however, aware of only one clear example where avian anti-predatory hissing calls have been shown to affect the behaviour of heterospecifics. Red-billed (*Buphagus erythrorynchus*) and yellow-billed oxpeckers (*B. africanus*) are adapted to exploit the arthropods that live on the skin of large herbivorous mammals (e.g. rhinoceros, Rhinocerotidae; buffalo, *Syncerus caffer*; giraffe, *Giraffa camelopardalis*), on which both species depend [141]. Interestingly, oxpeckers have long been believed to have a symbiotic defensive warning relationship with these large mammals and, in the Kiswahili language, oxpeckers are actually known as *Askari wa kifaru*, the ‘guard of the rhinoceros’ [141]. Specifically, oxpeckers produce hissing sounds when they detect the presence of a human (figure 1*i*; electronic supplementary material, video S11). A recent study revealed that red-billed oxpecker presence, number and hissing calling improved black rhinos’ (*Diceros bicornis*) human approach–detection rate and that it initiated rhino antipredator behaviours when they were found [142]. Hence, red-billed oxpeckers, by producing anti-predatory hissing calls, have been demonstrated to act as black rhinos’ anti-human sentinels.

Other evidence for the involvement of hissing signals in con- and/or heterospecific eavesdropping is inconclusive or indirect. For example, in geese, hissing by an individual often results in simultaneous hissing by pair or flock members [23], strongly suggesting that hissing can trigger collective anti-predatory responses. Also, it has been hypothesized that anti-predatory hissing calls of tit species could be used by migratory flycatchers in nest-site choice [143]. According to this ‘adaptive interspecific information use’ hypothesis, flycatchers obtain information about the perceived predation risk of nest cavities from, amongst others, hissing cues provided by tits, although evidence is currently lacking [143]. Moreover, within a mating context, both male and female black grouse likely eavesdrop on male hissing calls produced at leks in order to assess individual males (see §§5.5 and 6.1 [61]).

In general, con- and/or heterospecific eavesdropping on avian hissing signals is likely to be more widespread than currently evidenced (figure 2). It would hence be useful to broadcast anti-predatory hissing calls to conspecifics and/or sympatric heterospecifics and quantify their anti-predator response type and strength. Also, since animal communication typically occurs within a multiple signaller–receiver network, rather than being restricted to simple dyadic relationships [140], future studies of eavesdropping on hissing signals should explicitly adopt a network approach, especially in social and territorial avian species. This will further the understanding of whether and how (risk-related) information spreads throughout populations and animal communities, and hence communication network dynamics more generally.

## 6. Within- and across-population variation

Phenotypic variation typically occurs on different hierarchical levels, with variation occurring among taxa, among populations within species, and among individuals within populations. Phenotypic variation observed on all these levels can, in turn, have underlying genetic variation. In this section, we focus on summarizing evidence for variation in hissing sound production among individuals within and across populations of the same species and discuss potential underlying proximate mechanisms.

### 6.1. Repeatability and acoustic individuality

Over recent decades, it has been established that individuals within the same population can consistently differ in their behaviour across time and context, a phenomenon known as animal personality [144]. Animal personality specifically refers to among-individual differences in average behaviour across repeated observations [145] and the quantification of repeatability is pivotal in this regard, as it represents a standardized measure of variation among individuals and sets an upper limit to heritability [146]. Acknowledging this among-individual component of behaviour is important as it can play a key role in eco-evolutionary processes (e.g. biological invasion, population dynamics, predator-prey interaction, dispersal, response to human-induced rapid environmental change [147]) and can be linked with reproduction and survival [148–150].

Repeatability of hissing sound production has been demonstrated in a few avian species. In other words, within the same population, individuals have been shown to consistently differ in (aspects of) hissing behaviour. For example, in Canada geese, nest defence behaviour towards an approaching human, of which hissing is a part, has been shown to be short- (within year) and long-term (cross year) repeatable [94]. In little penguins, who can hiss as part of nest defence, individuals consistently differ in their response towards a simulated nest intrusion [151]. Moreover, when handled by a human observer, Eurasian kestrel nestlings consistently differ in their degree of agitation, which varies from not moving or calling during manipulation to struggling, flapping the wings and/or hissing all the time [91].

Further evidence for personality variation in hissing behaviour comes from great tits. That is, females, when confronted with a predator inside the nest cavity, consistently differ in the latency to produce hissing calls [54,87], whether or not they produce hissing calls [54,87,89,90] and the number of hissing calls produced during a 1-min predator intrusion test [88,152,153], altogether indicating that female great tits exhibit hissing behavioural types. Also, in other tit species, it has recently been demonstrated that female hissing behaviour can be repeatable [55,154] and hence that hissing behavioural types might be a common characteristic in tit populations. Despite previous attempts to elucidate the fitness costs and benefits associated with different hissing behavioural types (in great tits; [54,87–90,153]), it still remains puzzling to understand how this variation is maintained within natural populations (see §6.3).

In barn owl nestlings, acoustic features of hissing negotiation calls produced during sibling competition can to a large degree be attributed to individual nestlings (§5.4; [74]). That is, call rate and acoustic parameters of calls (related to duration, frequency, loudness and within-call variation) were all found to be repeatable. Based on acoustic parameters, nestlings could also be (statistically) discriminated from their siblings, indicating that a nestling produced hissing calls that were consistently similar in their structure. Moreover, a cross-fostering experimental design revealed that most of the acoustic features related to the nest of origin, but none to the nest of rearing, suggesting a genetic or early developmental effect on the ontogeny of individually distinctive acoustic characteristics (§6.2). Altogether, this suggests that barn owl nestlings might be able to recognize their siblings individually based on aspects of hissing signals [74].

Individual recognition is part of many social interactions observed in nature, and signals that evolve to facilitate individual recognition are called identity signals [155]. Vocal individuality, and hence vocal identity signalling, is believed to be a common feature of many vocally active species [155–157]. Individual recognition based on vocal identity signals has been shown to be important in a range of social contexts, including territoriality, aggressive competition, cooperation, antipredator behaviour, foraging behaviour, parent-offspring recognition and mate recognition (reviews in [155,157–159]). Despite omnipresent evidence for vocal individuality in a variety of taxa, including birds, the possibility of individuality in non-vocal acoustic signals has received very limited research attention.

Acoustic individuality in avian hissing sounds has, to our knowledge, only been investigated by three studies. First, as described above, nestling barn owl hissing negotiation calls during sibling competition are to a large degree individual-specific, which is believed to be advantageous in maintaining honesty in this sib–sib communication system [74]. Second, the domestic goose produces hisses when threatened, and a variety of features of such hissing calls are individually distinctive [23]. In this case, it has been argued that individual recognition of hissing mates in dangerous situations may increase the probability of their survival via a more efficient anti-predator response (see also [158]). Third and finally, in the black grouse, where males use long-distance hissing calls during mating displays at leks (§5.5), it was demonstrated that, based on acoustic parameters, hissing calls could be attributed to individual males [61].

As apparent from these examples, non-vocal hissing signals may encode more information than previously thought, including the caller’s identity. Although studies are too few to draw solid conclusions, it is likely that sociality is an important driver of non-vocal acoustic individuality, as has been hypothesized and demonstrated for vocal and other (visual, olfactory) identity signals [155–157]. Interestingly, other examples of non-vocal acoustic signals that can encode individual identity include drumming in territorial great spotted woodpeckers (*Dendrocopos major* [7]) and mechanically produced ‘swishes’ during wing-rustling in lekking male greater sage-grouse (*Centrocerus urophasianus* [160]). Hence, it can be argued that the evolution of individuality in non-vocal acoustic signals could generally be expected in social or territorial species, especially those with a high rate of (repeated) social interactions and/or complex social systems.

Although individuals of a species may produce distinctive hissing sounds, receivers may not recognize the caller as unique. Complex (sensory and cognitive) mechanisms for individual recognition are expected to evolve only when simpler mechanisms do not prove sufficient to obtain the available advantages, in terms of reproduction and survival in a particular environment [159]. To date, no study has explicitly assessed whether receivers discriminate among individuals based on hissing signals and direct evidence for hissing-based individual recognition is thereby lacking. Assessing individual recognition for hissing signals, as for any signal, would require an experimental approach, such as playback experiments, go no-go experiments, discrimination tasks or habituation/dishabituation experiments (see [157] for full discussion). Ideally, experiments are designed in a way that it can be determined whether a focal individual can recognize multiple individuals based on the (identity) signal(s) of interest [157,159].

Overall, future research should explicitly assess among-individual variation in hissing signals while incorporating a number of previously overlooked aspects. That is, although many studies have observed or quantified (aspects of) hissing behaviour in a variety of avian species (electronic supplementary material, table S1; §7), very few have used a repeated measurement design to quantify repeatability. The latter is important as repeatability sets an upper limit to heritability, and hence underlying genetic variation and the potential to adaptively evolve under selection [146]. Moreover, we are unaware of any study assessing temporal consistency of individuality in hissing signals. Repeated measurements on individuals should be obtained within social contexts and across ecologically relevant timescales for the species of interest, allowing solid tests of both the repeatability and the stability of individuality and individual recognition. At the same time, studies on individual recognition should explicitly focus on both the signaller (i.e. the [dis]advantage of being recognized) and the receiver (i.e. the [dis]advantage of individually recognizing conspecifics [155,157]).

### 6.2. A proximate perspective

Phenotypic variation in any given trait may be shaped by a mix of genetic, environmental (physical, social) and developmental factors [161]. To date, we are aware of only one study investigating the heritability of hissing signals. As mentioned above, a cross-fostering design in barn owls was used to estimate the heritability of acoustic features of nestling hissing negotiation calls. Individual variation in call duration, loudness, loudness deviation (i.e. within-call variation in amplitude) and mean frequency were all found to be heritable, suggesting a genetic or early developmental effect for these features [74]. An important step forward would be to use a quantitative genetics approach, thereby enabling the partitioning of the observed phenotypic variation in hissing behaviour into its underlying genetic and non-genetic components [161].

At the same time, very little is known about the specific genes underlying individual variation in avian hissing sound production. One notable exception comes from the anti-predatory hissing behaviour in female great tits (§3). Using a candidate gene approach, a series of recent studies revealed that individual variation in hissing behaviour can be linked to single nucleotide polymorphisms in the serotonin transporter gene [162–165], but not the dopamine-receptor D4 gene [162]. The serotonin transporter plays an important role in the regulation of extracellular and synaptic serotonin concentrations, and hence the magnitude and duration of serotonergic neurotransmission. Interestingly, serotonin might have an evolutionary well-conserved function in hissing behaviour, given it has also been shown to be involved in the modulation of territorial hissing in lizards [166] and defensive hissing in domesticated cats [167]. Yet, the exact neuroendocrine bases and causal physiological mechanisms underlying variation in avian hissing sound production remain virtually unexplored.

Since many avian species produce hissing signals in challenging situations, such as predator confrontation and/or agonistic interactions, it can be hypothesized that individual variation in hissing behaviour can be part of an individual’s coping style [168]. Following this line of research, it can be predicted that repeatable variation in the expression of hissing behaviour is linked with individual variation in a range of (neuro)physiological mechanisms, such as stress responsiveness, androgen levels, (para)sympathetic reactivity and serotonin and dopamine systems [168–170]. As outlined above, only for serotonin is there currently some evidence with regard to anti-predatory hissing behaviour in female great tits.

Clearly, research on the genetics, neuroendocrine bases and causal physiological mechanisms underlying individual variation in avian hissing sound production is still in its infancy. Hissing signals expressed in different social/behavioural contexts (e.g. predator confrontation, mating context) may, or may not, be influenced by different causal (neuro)physiological mechanisms. Also, seasonal variation and sex differences in hissing sound production deserve specific attention from a proximate perspective, especially given that hissing call production can be closely linked with the annual reproductive cycle and hence hormonally mediated reproductive activities, including courtship-related sound production (§5.5). Recent tools available for quantitative and molecular genetics [171,172], combined with the investigation of (neuro)physiological underpinnings, will prove particularly helpful in revealing the heritability of, and proximate mechanisms underlying, individual variation in avian hissing sound production in different contexts, seasons and sexes.

### 6.3. Geographic variation

Phenotypic (behavioural) variation across populations of the same species can arise due to spatio-temporal variation in selection caused by local environmental factors such as weather conditions, habitat characteristics, (breeding) density, predation pressure, food availability and human disturbance levels [86,173–175]. Previous research has mainly focused on geographic variation in the vocal performance (i.e. song) of vocally learning bird species (see [176]), while non-vocal acoustic signals only recently attracted some research attention. Evidence for cross-population variation in (aspects of) hissing behaviour is hence scarce but exists for certain avian study systems.

In the black grouse, hissing calls from populations in four different European countries were found to show geographic variability. Specifically, male black grouse could largely be attributed to different populations based on a variety of frequency, amplitude and temporal features of hissing calls produced [62]. In little penguins, nest defence, of which hissing is a part, was found to be higher in an island population with higher unregulated human disturbance compared to a population with lower disturbance [151]. In cinereous tits (*Parus cinereus*), anti-predatory hissing behaviour showed latitudinal variation across China, with females breeding at lower latitudes (more southern populations) being less likely to produce hissing calls upon predator confrontation compared to females breeding at higher latitudes (more northern populations) [55]. Also, in Japanese tits (*Parus minor*), predation was shown to affect hissing behaviour, with the occurrence of hissing displays across populations being dependent on a combination of both predator type and local predation risk level [177]. Finally, in great tits, the proportion of females producing anti-predatory hissing displays has been found to be higher in coniferous forests (compared to deciduous forests; [89]), at lower breeding densities [87] and in populations where snakes were more abundant [90]. In contrast, the occurrence of hissing displays was not found to differ between urban and rural breeding great tits [178].

As apparent from these examples, direct evidence for the underlying selective drivers of geographic variation and/or population differences in hissing sound production remains very scarce. Further research should therefore focus on investigating aspects of hissing sound production (e.g. occurrence, frequency, spectrographic features) in different populations across geographic scales relevant for the species of interest. This would enable us to establish whether: (i) geographic variation in non-vocal hissing signals is common across populations; (ii) local dialects exist for non-vocal acoustic signals, as is often found to be the case for bird song [176]; and (iii) variation in hissing behaviour comes with fitness consequences resulting from geographic and/or temporal variation in habitat and population-specific selection pressure(s). Investigating the latter requires long-term (across years) population monitoring, during which behavioural, fitness (e.g. reproductive output, survival), ecological and demographic data should be obtained within and across populations.

## 7. Phylogeny of avian hissing sound production

Birds (Aves) are composed of two sister clades, Paleognathae and Neognathae [78,179,180]. The extant group of paleognaths is composed of about 60 species, while there are more than 10 000 species of extant neognaths [13]. Also within the neognaths, two sister clades exist, the Galloanserae (water- and landfowl) and the Neoaves (all other living neognaths [179,180]). Given this extraordinary number of extant species, combined with rather disparate published accounts on hissing sound production, we do not aim to give a full overview of species that produce hissing sounds. Additionally, the lack of accurate absence data on hissing sound production prevented us from performing a detailed phylogenetic analysis. Therefore, we instead focus on summarizing evidence for hissing in the taxonomic avian orders and give non-exhaustive examples of families and species to illustrate (electronic supplementary material, table S1).

### 7.1. Paleognathae

The Paleognathae consist of the flightless ratites (ostriches [Struthioniformes], rheas [Rheiformes], kiwi [Apterygiformes], emus and cassowaries [Casuariiformes]) and the volant tinamous (Tinamiformes). The ostriches are a sister taxon to all other extant paleognaths and hence take an influential phylogenetic position in estimating ancestral states in Paleognathae [78]. The ostrich is well-known for producing hissing calls, where both adult males and females produce various open-mouthed hissing sounds during aggression [25,40]. Members of both sexes of the Rheiformes, Apterygiformes, and Casuariiformes are also known to produce hissing calls when agitated (electronic supplementary material, table S1 [40,81,181]), indicating that hissing sound production is present in all four orders of ratites.

In tinamous, there appear to be no primary accounts of hissing sound production, despite a number of recent reports dealing with the acoustic behaviour in various species (e.g. [182–184]). However, given their often secretive lifestyle, much remains unknown about even their basic natural history. Solid experimental investigation, for example, by conspecific or heterospecific simulated (territory) intrusion tests, is therefore necessary to establish whether or not species of tinamou produce hissing sounds.

### 7.2. Neognathae

#### 7.2.1. Galloanserae

The Galloanserae form the basal clade within the neognaths and are composed of two orders, the Anseriformes (waterfowl) and the Galliformes (landfowl [180]). The Anseriformes includes ducks, geese, swans and screamers. Members of the family Anatidae are well-known to hiss during territory and nest defence, as demonstrated by quantitative studies on both sexes of geese, swans and ducks (electronic supplementary material, table S1). Also, for the sole extant member of the family Anseranatidae, the magpie goose (*Anseranas semipalmata*), there is evidence that hissing occurs during nest defence [185]. In contrast, for the family Anhimidae (screamers), which consists of three extant species, primary reports on their acoustic behaviour appear to be lacking.

The Galliformes is composed of five families. Within this order, by far the most evidence for the production of hissing sounds comes from members of the family Phasianidae (electronic supplementary material, table S1). Species include the black grouse, ruffed grouse and tragopan species, all of which have been described to hiss during mating displays (§5.5). Additionally, some species of this family produce hissing calls in response to (predator) threat, including the ring-necked pheasant (*Phasianus colchicus*), grey partridge (*Perdix perdix*), willow ptarmigan (*Lagopus lagopus*), Indian peafowl and red junglefowl (*Gallus gallus*; electronic supplementary material, table S1). Primary accounts for hissing in the other four families of Galliformes are very scarce. That is, no reports appear to be available for either the Megapodiidae (megapods), Cracidae (guans, chachalacas and curassows) or Odontophoridae (new world quail). In contrast, at least one report exists for the family Numididae (guineafowl), where females of the helmeted guineafowl (*Numida meleagris*) emit hissing sounds during nest defence [186].

As apparent, the production of hissing sounds is widespread among early-branching avian lineages. Specifically, hissing sound production can be found in at least six of seven orders and is represented in at least eight families within the paleognaths and basal neognaths (Galloanserae).

#### 7.2.2. Neoaves

The Neoaves encompasses 95% of extant avian species and a significant proportion of their phylogenetic diversity. Specifically, following [13], Neoaves comprises 238 families within 34 orders. In the following, we discuss some particularly noteworthy observations of hissing sound production within the Neoaves, while a full list of orders and families can be found in electronic supplementary material, table S1.

The Caprimulgiformes (or Strisores) defines a basal radiation of Neoaves [180]. Within the family Caprimulgidae (nightjars), at least ten species have been described to produce hissing sounds during threat/defence displays (electronic supplementary material, table S1). In the family Podargidae (frogmouths), nestlings and adults of at least two species hiss during threat/defence displays [63]. Moreover, the Australian owlet-nightjar (family Aegothelidae) has been shown to hiss as part of threat display [36,67]. In addition, anecdotal evidence exists that nestlings of the white-rumped swift (*Apus caffer*; family Apodidae) very occasionally produce guttural hisses [187]. Hence, hissing occurs in members of at least four families of the Caprimulgiformes, indicating it is widespread in this basal radiation of Neoaves.

The Opisthocomiformes consist of one family (Opisthocomidae) with a sole representative, the hoatzin (*Opisthocomus hoazin*). In this species, the usual nest defence and alarm call has been described as a raspy hiss, a high-intensity nasal wheezing, which is uttered in response to predators, human disturbance and during low-level territorial interactions [70]. Also, the Eurypygiformes is composed of two families, each with a sole representative. In the family Rhynochetidae, the kagu (*Rhynochetos jubatus*) hisses in response to predators [188] and in the family Eurypygidae, the sunbittern (*Eurypyga helias*) is known to produce hissing calls during nest defence displays [189].

Within the Gruiformes, hissing has been reported for members of at least three families. For example, the white-winged flufftail (*Sarothrura ayersi*; family Sarothruridae) and the Galápagos rail (*Laterallus spilonota*; family Rallidae) hiss when disturbed at the nest [190,191]. Furthermore, probably all 15 species of the family of cranes (Gruidae) produce hissing calls when attacking and mobbing intruders (electronic supplementary material, table S1 [192]).

The Charadriiformes is a large avian order including shorebirds and relatives. Despite the relatively large number of species within this order, remarkably few primary accounts appear to exist on the production of hissing sounds. Yet, hissing sound production has been described in the Eurasian thick-knee (*Burhinus oedicnemus*; family Burhinidae), the greater painted-snipe (*Rostratula benghalensis*; family Rostratulidae), the parasitic jaeger (family Stercorariidae) and both the black (*Cepphys grylle*) and pigeon guillemot (*C. columba*; family Alcidae; electronic supplementary material, table S1).

In the Cathartiformes, with the sole family Cathartidae (New World vultures), numerous accounts exist on the occurrence of hissing sound production. For example, in the turkey vulture (*Cathartes aura*), nestlings and adults produce an inhalant hissing sound whenever disturbed (figure 1*e*; [193,194]). Also, nestlings and adults of the black vulture (*Coragyps atratus*) have been observed to produce hissing sounds upon disturbance [195]. Moreover, in the Andean condor (*Vultur gryphus*), soft and quiet hisses are part of courtship display [196].

The Accipitriformes is an order composed of raptors. Within the family Accipitridae (hawks, eagles and kites), various members were found to produce hissing sounds. The tiny hawk (*Accipiter superciliosus*) has been observed to produce hissing sounds towards intruders [197]. Anecdotal evidence also suggests that golden eagle (*Aquila chrysaetos*) nestlings hiss softly in response to a human at the nest [45] and nestlings of the white-tailed kite (*Elanus leucurus*) similarly produce hissing sounds when disturbed [198]. Furthermore, palm-nut vultures (*Gypohierax angolensis*) produce a hissing whistle during mating [199] and Eurasian griffon vultures hiss during intraspecific food competition [99].

The Strigiformes is composed of two families, the Tytonidae (barn owls) and Strigidae (owls) and numerous members of both families produce hissing sounds. Within the Tytonidae, nestling and adult barn owls are well-known to utter hissing calls under threat [45,66] and nestlings utter hiss-like negotiation calls during sibling competition and parental feeding [108]. Also, the Sulawesi masked-owl (*Tyto rosenberghii*) and the African grass-owl (*Tyto capensis*) produce hissing sounds (electronic supplementary material, table S1). Within the Strigidae, burrowing owls hiss when cornered in their burrow [33]. Likewise, many other species of Strigidae perform bill-snapping and non-vocal defensive hisses when cornered, including, for example, the Eastern screech owl (*Megascops asio*), short-eared owl (*Asio flammeus*) and great horned owl (*Bubo virginianus*; review in [33]).

The Bucerotiformes consist of four families. Within the family Upupidae (hoopoes), nestling and adult Eurasian hoopoes emit hissing sounds upon predator confrontation [80]. Similarly, within the family Phoeniculidae (wood hoopoes and scimitarbills), nestlings and adults of the common scimitarbill produce hissing sounds when disturbed inside the nest cavity [79]. Within the family Bucorvidae (ground-hornbills), evidence exists that nestling Southern ground-hornbills (*Bucorvus leadbeateri*) hiss when confronted with a human observer at the nest [200]. Finally, within the family Bucerotidae (hornbills), female writhe-billed hornbills are known to produce a subdued hissing sound in response to male feedings at the nest (§5.6; [138]).

In the Psittaciformes (parrots), various members, when frightened and cornered without possibility of escape, fluff the body and head feathers, lift the wings partly away from the body and rock slightly from side to side, generally hissing and sometimes lunging with the bill (figure 1*g*). They may also bow up and down (electronic supplementary material, video S12; [201]). This display is typically found within the Cacatuidae (cockatoos) and Psittacidae (New World parrots; electronic supplementary material, table S1; [201]). Also, when threatened, brush-tongued parrots (lories and lorikeets; Psittaculidae) bow and hiss [202,203]. Moreover, in many species of lorikeets, nestlings produce hissing sounds when begging for food from their parents [109].

The Passeriformes (perching birds) are the largest avian order with over 6000 extant species in 143 families [13]. As mentioned before, the best-known examples of hissing sound production come from members of the family of Paridae, with primary accounts for at least 17 species (electronic supplementary material, table S1). Apart from the Paridae, published accounts of hissing sound production appear to be sparsely scattered across families within the Passeriformes. Within the suborder Suboscines (Tyranni), hissing sound production can notably be found in members of the Thamnophilidae (antbirds), Furnariidae (woodcreepers and allies) and Tyrannidae (tyrant flycatchers; electronic supplementary material, table S1). Within the suborder Oscines (Songbirds), members that produce hissing sounds can be found in, amongst others, the Ptilonorhynchidae (bowerbirds), Paradisaeidae (birds-of-paradise), Corvidae (crow, jays and magpies), Phylloscopidae (leaf warblers), Troglodytidae (wrens), Ploceidae (weavers and allies) and Passerellidae (New World sparrows; see electronic supplementary material, table S1 for full list).

In various avian orders not discussed before, only a single or few members were found to produce hissing sounds. This includes the Podicipediformes (grebes), Columbiformes (pigeons and doves), Otidiformes (bustards), Musophagiformes (turacos), Cuculiformes (cuckoos), Procellariiformes (tube-nosed seabirds), Coraciiformes (kingfishers and allies) and Piciformes (woodpeckers and allies; electronic supplementary material, table S1). On the other hand, in a variety of avian orders not discussed before, hissing sound production appeared to be more widespread, such as in the Sphenisciformes (penguins), Ciconiiformes (storks), Suliformes (totipalmate water and diving birds), Pelecaniformes (ibis, herons, pelicans, hammerkop and shoebill), Trogoniformes (trogons) and Falconiformes (falcons; electronic supplementary material, table S1). Overall, our literature review reveals that, across both the Paleognathae and Neognathae, hissing sound production is present in members of at least 34 avian orders and at least 86 families (electronic supplementary material, table S1).

## 8. Conclusions and future directions

From this review, it is apparent that avian hissing sounds are widespread and can be involved in many aspects of avian life, including predator–prey dynamics, agonistic interactions, family dynamics (i.e. parent-offspring communication, sibling competition and negotiation), mating behaviour, pair communication during the breeding season and con- and/or heterospecific eavesdropping. Moreover, accumulating, although still very limited, evidence indicates that variation in (aspects of) hissing sounds can be repeatable within individuals, encode individual identity, have underlying genetic variation and may come with fitness consequences. Altogether, this emphasizes that research on avian hissing sounds should be firmly integrated into many crucial and contemporary disciplines of behavioural and evolutionary ecology.

Our understanding of avian hissing sounds can be vastly improved by the investigation of several previously largely overlooked aspects. First, despite being hypothesized as a basal and primitive sound production mechanism, almost nothing is currently known about the mechanics of hissing sound production in birds. Insights into the exact mechanism(s), including the determination of the location(s) of mechanistic constriction along the vocal tract and potential syringeal involvement, will provide essential clues to the anatomy, ontogeny and evolution of avian hissing sound production. At the same time, it will specifically help in determining the occurrence of non-vocal and/or vocal hissing sound production in the variety of avian species that emit them.

Second, (comparative) spectrographic analyses are necessary to determine spectral, temporal and amplitude features of avian hissing sounds within and across populations and species. This will not only provide the necessary data to investigate whether and how hissing sounds are distinct on the individual-, population- and species-level, but also enable the investigation of the (dis)similarity of bird hisses and snake hisses, and hence the occurrence of acoustic snake mimicry.

Third, carefully designed playback experiments are essential to firmly establish whether and how naturally occurring predators and heterospecific/conspecific competitors respond to avian hissing sounds. This will enable us to determine the exact function(s) of hissing signals, which are currently obscure or merely anecdotal for many avian species (electronic supplementary material, table S1). In the case of anti-predatory hissing, the playback of hissing signals of both birds and snakes to predators will additionally help in establishing the occurrence of acoustic snake mimicry. Also, it would be useful to broadcast hissing sounds to conspecifics and sympatric heterospecifics to investigate the occurrence of (anti-predatory) eavesdropping, preferably within a social network approach. Moreover, playback experiments in species with individually distinct hissing sounds are necessary to determine whether individual recognition occurs based on hissing (identity) signals.

Fourth, performing con- and/or heterospecific simulated (territorial) intrusion tests (e.g. by using dummies) is pivotal to obtain quantitative and qualitative data on hissing sounds. Ideally, a repeated measurement design is employed to obtain the necessary data to quantify the repeatability of hissing behaviour. Such a design, combined with spectrographic analyses, will also be useful in determining the temporal stability of individuality in hissing signals within contexts and across relevant temporal scales for the respective avian species.

Fifth, solid investigations into the underlying genetics and proximate mechanisms of individual variation in hissing sound production are due. Employing repeated measurement designs in pedigreed populations will provide the necessary data for a quantitative genetics approach, and hence the quantification of the heritability of hissing behaviour. A molecular genetics approach can, in turn, be used to identify the specific genes involved in the expression of individual variation in hissing behaviour. Moreover, individual variation in the expression of hissing behaviour should be firmly integrated into coping style research, thereby aiming to reveal variation in underlying (neuro)physiological mechanisms (e.g. stress responsiveness, androgen levels, (para)sympathetic reactivity, neurotransmitter systems).

Sixth, all the research methods outlined above should explicitly consider the potential for sex and age effects, since currently, there are many unresolved questions regarding sex differences and the development of hissing sound production. For example, in ratites, why do adults of both sexes produce hissing sounds, while juveniles do not, and at what developmental stage do they acquire this ability? Why are hiss-like begging calls rather widespread, while adults of many of these species do not appear to produce hissing sounds? Are there species in which (only) females produce hissing sounds within a mating context, and how common are sex differences in the occurrence, frequency and/or acoustic features of hissing sounds in other contexts? More generally, since the ability to produce hissing sounds is most likely the ancestral condition [3], which (anatomical, developmental and/or ecological) factors drive the secondary loss in one of the sexes and/or across age? Together, this emphasizes the need for future studies on avian hissing sounds to integrate functional morphology, neurobiology, physiology and bioacoustics across sexes and development.

Seventh, future research should focus on evaluating the fitness consequences of individual variation in hissing sound production in terms of mating success (in the case of mating displays), reproductive success and survival in the wild. Pinpointing exact selection pressures on hissing signals can be done by monitoring populations in the long run (i.e. across years) and/or comparing populations that are known to differ in selective regimes due to (local) environmental factors. Combined knowledge on heritability and selection pressure(s) will be necessary to determine the potentially adaptive evolution of individual variation in hissing behaviour.

Finally, we firmly established that avian hissing sounds can be involved in both inter- and intraspecific communication and that their functions span many crucial aspects of avian life. This is in contrast to evidence in non-avian reptiles, where hissing sounds are generally not believed to be involved in intraspecific communication, but only serve an interspecific (i.e. threat/defence) function ([15,17]; see also [12]). This raises the intriguing hypothesis that birds have evolved a whole (new) set of functions for hissing sounds within intra-specific communication, potentially co-evolved with better acoustic sensitivity (i.e. hearing ability) in birds compared to many non-avian reptiles [204]. This hypothesis warrants further investigation as it opens a debate about the use and evolutionary origin of broadband hissing sounds in intraspecific acoustic communication.

## Data Availability

Data associated with this article can be found in the supplementary material [205].
